# The gut-brain-axis one year after treatment with cladribine tablets in patients with relapsing remitting multiple sclerosis: a pilot study

**DOI:** 10.3389/fimmu.2025.1514762

**Published:** 2025-02-27

**Authors:** Jeske van Pamelen, Carla Rodriguez-Mogeda, Lynn van Olst, Susanne M. A. van der Pol, Maarten L. Boon, Janet de Beukelaar, Oliver H. H. Gerlach, Andries E. Budding, Joep Killestein, Helga E. de Vries, Leo H. Visser

**Affiliations:** ^1^ Department of Neurology, Elisabeth-TweeSteden Hospital, Tilburg, Netherlands; ^2^ Multiple Sclerosis (MS) Center Amsterdam, Amsterdam Neuroscience, Amsterdam University Medical Center (UMC) Location Vrije Universiteit Amsterdam, Amsterdam, Netherlands; ^3^ Department of Molecular Cell Biology and Immunology, MS Center Amsterdam, Amsterdam Neuroscience, Amsterdam UMC Location Vrije Universiteit Amsterdam, Amsterdam, Netherlands; ^4^ inBiome, Amsterdam, Netherlands; ^5^ Department of Neurology, Albert Schweitzer Hospital, Dordrecht, Netherlands; ^6^ Department of Neurology, Zuyderland Medical Center, Sittard-Geleen, Netherlands; ^7^ School for Mental Health and Neuroscience, Maastricht University, Maastricht, Netherlands

**Keywords:** relapsing remitting multiple sclerosis, cladribine tablets, gut-brain-axis, microbiota, mass cytometry by time-of-flight, 16S-23S rDNA interspace profiling

## Abstract

**Introduction:**

Cladribine tablets are an effective treatment for relapsing remitting multiple sclerosis (RRMS). However, almost half of the treated patients are not free of disease activity after two years. The aim of this study was to describe the changes that cladribine tablets effectuate in the gut and oral microbiota and the peripheral immunological profile between responders and non-responders.

**Methods:**

In this pilot study of the multicenter, prospective, observational BIA (Brain-Immune-Intestine Axis) study, we included patients aged 18 to 55 years with RRMS who were scheduled to start treatment with cladribine tablets. We assessed the clinical status and the immunological and microbiological profile prior to the start of the treatment and after three and twelve months. At twelve months, we assessed the response status, based on clinical relapses, radiological activity and disability progression on the Expanded Disability Status Scale.

**Results:**

The first twenty-five patients of the BIA study were included in this analysis. Ten patients (40%) were responders twelve months after treatment. Three months after treatment we found a significant decline of naïve and transitional B cells and memory B cells, and of CD57^+^ CD56^dim^ NK cells. After twelve months the values recovered to baseline levels, except for the memory B cells. We did not find significant changes of the microbiological profile over time, except for a decline of the phylum *Bacteroidetes* in the oral samples twelve months after treatment. Baseline values and changes over time did not significantly differ between responders and non-responders. However, several phyla, genera or species (*Bacteroidetes, Prevotella, Faecalibacterium prausnitzii)* showed a higher relative abundance, and several phyla, genera or species (*Proteobacteria, Escherichia coli)* had a lower relative abundance in responders compared to non-responders.

**Discussion:**

After treatment with cladribine tablets, we found significant changes in the immunological landscape. Also, the microbiological profile showed several differences in microbes with known anti- or pro-inflammatory properties between responders and non-responders. Overall, we showed that we can measure a treatment effect from cladribine tablets with our analyses. Future research on data from the BIA study, with a larger sample size and extended follow-up, can possibly confirm the reliability of our findings.

## 1 Introduction

Cladribine tablets are an effective treatment in patients with relapsing remitting multiple sclerosis (RRMS). However, almost half of the treated patients are not free of disease activity after two years (1). The reason for these individual treatment responses is not understood yet. An effective treatment is important to achieve or maintain a stable disease status, and potential side effects are accepted if the potential benefit outweighs the risks of the treatment. However, at the moment it is not possible to predict which patient will benefit from cladribine treatment. This results in the exposure of patients to possible harmful side effects, without the desired effect on disease activity. One of the factors underlying the individual treatment response could be the interaction between the gut, the brain and the immune system, also called the gut-brain axis or the brain-immune-intestine axis. A better understanding of this gut-brain axis and the changes that cladribine tablets effectuate can potentially help to unravel why an individual patient becomes a responder or a non-responder.

Cladribine is a synthetic deoxyadenosine analogue, which is registered in its oral form for the treatment of highly active RRMS (2, 3). Oral cladribine is an immune reconstitution therapy, which is administered during two treatment weeks in the first year and two treatment weeks in the second year (2). After the second treatment course, no further treatment should be required in year three and four. In a randomized controlled trial, 44% of the treated patients had no evidence of disease activity (NEDA-3) after 96 weeks, which means that these patients did not experience relapses, showed no disability progression and did not have radiological disease activity on MRI (1). The therapeutic effects of cladribine are attributed to selective depletion of immune cells. Several studies described a marked decline of B lymphocytes and a modest decline of both CD4^+^ and CD8^+^ T lymphocytes (4–9).

The immune system is an important player in the pathogenesis and the treatment of multiple sclerosis (MS) and is mediated by several factors. Several studies on experimental autoimmune encephalomyelitis (EAE), an animal model of MS, have shown that the commensal gut flora is essential in triggering immune processes, as germ-free mice developed less severe EAE or no EAE at all (10, 11). Furthermore, mice that received intestinal bacteria from patients with MS (pwMS) showed a higher frequency of EAE or more severe EAE than mice that were colonized with intestinal bacteria from healthy persons (12, 13). These changes in the frequency or intensity of EAE were accompanied by changes in the microbiota or the immune profile, or both, which provides some evidence for the importance of the gut-brain axis in EAE (11–13).

Following the findings in EAE, the gut-brain axis in pwMS has also been studied extensively. Several studies found that the gut microbiota in pwMS differ from the gut microbiota in healthy controls, and that pwMS had either a lower number of immunomodulatory cells, such as regulatory T cells, or a higher number of pro-inflammatory cells, such as Th1 and Th17 lymphocytes, or both, compared to healthy controls (14–26). Studies that investigated the differences in the microbiota between MS patients with and without immunomodulatory treatment (IMT) revealed different composition of the microbiota (15, 18–20, 25, 27). Furthermore, several longitudinal studies showed changes in the abundance of numerous bacterial phylae or species following treatment with dimethyl fumarate and ocrelizumab (21, 28–30). Other studies were able to show a correlation between the levels of immune cells and the abundance of different bacterial species (22–24, 31). Although the gut-brain axis has been studied extensively, the oral-brain axis in neurological diseases is an underexposed area. While it is relatively easy to obtain oral samples, and the microbial compositions of oral and fecal swabs share a degree of consistency, only a few studies investigated the potential role of the oral microbiota in the pathophysiology of MS (32, 33). Several animal studies provided insights in the potential link between the oral microbiota and MS, and several clinical studies found differences in genera and phyla levels between pwMS and healthy controls (33).

Data on the microbiota changes after treatment with cladribine are limited. Also, there is a lack of research trying to understand the correlation between changes in the microbiota, the immune system and treatment responses.

Hence, the aim of this pilot study was to describe both the changes in gut and oral microbiota and immunological profile three and twelve months after treatment with cladribine tablets, and to determine if the composition of the gut and oral microbiota and the immunological profile differs between responders and non-responders to the treatment.

## 2 Methods

### 2.1 Population

Data were used from the multicenter, prospective, observational BIA (Brain-Immune-Intestine Axis) study (34). The BIA study recruited patients from eleven outpatient clinics in the Netherlands and Belgium between January 2019 and October 2022. Patients were asked to participate in the study if they had relapsing remitting multiple sclerosis (RRMS) and were scheduled to start with cladribine tablets as per standard of care. Participants had to be between the age of 18 and 55 years, and were excluded if they had used probiotics within one month prior to the planned start of cladribine. For this pilot study, we selected the first 25 patients who had completed the baseline visit and the follow-up visit at three months, and who were not missing peripheral blood mononuclear cell (PBMC) samples and microbiological samples for both baseline and month three follow-up visits. If available, the data of their follow-up visit at twelve months were also used for this analysis.

### 2.2 Study procedures

**Figure 1:**
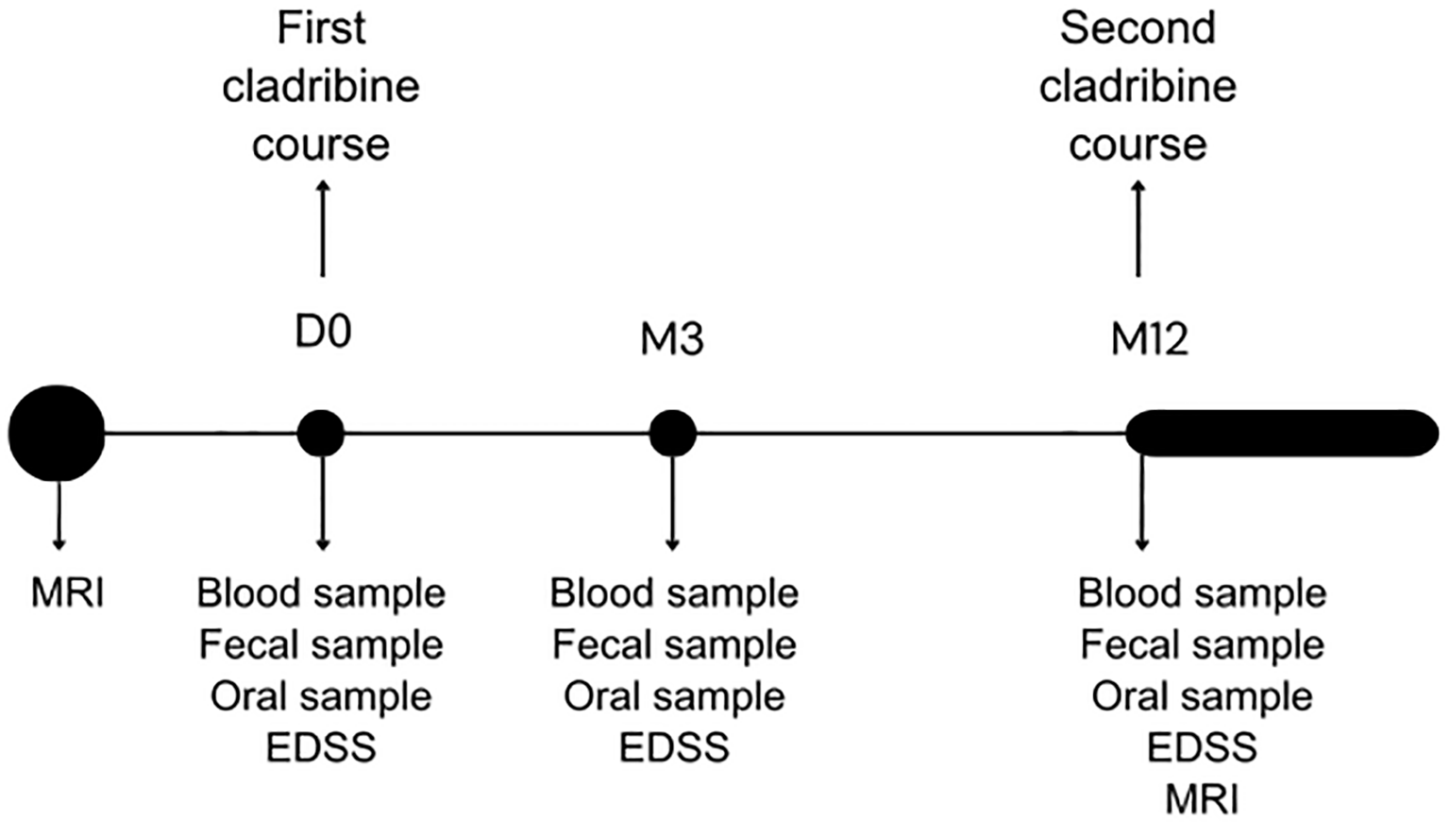
Study timelines. D0: baseline visit. M3: follow-up visit after 3 months. M12: follow-up visit after 12 months. M12 takes places before start of the second cladribine course and can be postponed until M18.

For each patient, a total of three study visits were carried out in a period of one to one and a half year. The first study visit was performed within one week prior to the start of cladribine tablets (baseline visit, D0). Next, two follow-up visits took place: three months after the first course of cladribine tablets (M3) and prior to the start of the second course of cladribine tablets (which is normally twelve months after the first course, but can be delayed for up to six months if medically necessary, M12). In case of a relapse, an extra visit was scheduled within 14 days from onset to obtain a score on the Kurtzke Expanded Disability Status Scale (EDSS) (35). The study timeline is displayed in **Figure 1**.

During each visit we determined the score on the EDSS and obtained a blood sample, a fecal sample and an oral sample. As per standard of care, an MRI of the brain was performed within three months before start of cladribine tablets and twelve months after the first treatment course. More detailed information on collected data was published in the research protocol (34).

For this pilot study, we used the available data and samples from D0, M3, M12 and relapse visits, and collected the results of brain MRIs that were performed as per standard of care within the period between start of the first treatment course of cladribine tablets and the M12 visit.

### 2.3 Outcome measures

#### 2.3.1 Immunological profile

Blood was collected for isolation of PBMCs using Vacutained Mononuclear Cell Preparation Tubes with sodium citrate (CPT, BD Biosciences). PBMCs were stored in -80°C and within three months after collection in liquid nitrogen until further staining and analysis for mass cytometry by time-of-flight (CyTOF). PBMCs were stained using the MaxPar Direct Immune Profiling Assay (Standard BioTools) following the manufacturer’s instructions and as similarly published, but with some modifications (**Table 1**) (36, 37). We first barcoded the samples using CD45 barcoding mixes (Standard BioTools). After staining with MaxPar Direct Immune Profiling Assay, cells were incubated with MaxPar Intercalator-Ir (1:000 diluted in MaxPar Fix and Perm, Standard BioTools) for one hour. Cells were counted and frozen in cryovials containing 5x10^6^ cells in MaxPar Intercalator-Ir solution. Within each CyTOF staining and acquisition, one reference sample was included to be able to normalize for staining intensity differences between batches.

**Table 1:** Antibodies for CyTOF.

Markers	Metals
CD45	89Y
CD196/CCR6	141Pr
CD123	143Nd
CD19	144Nd
CD4	145Nd
CD8a	146Nd
CD11c	147Sm
CD16	148Nd
CD45RO	149Sm
CD45RA	150Nd
CD161	151Eu
CD194/CCR4	152Sm
CD25	153Eu
CD27	154Sm
CD57	155Gd
CD183/CXCR3	156Gd
CD185/CXCR5	158Gd
CD28	160Gd
CD38	161Dy
CD56/NCAM	163Dy
TCRgd	164Dy
CD294	166Er
CD197/CCR7	167Er
CD14	168Er
CD3	170Er
CD20	171Yb
CD66b	172Yb
HLA-DR	173Yb
IgD	174Yb
CD127	176Yb
Live/dead intercalator	103Rh

During the days of acquisition, each cryovial was defrosted and cells were acquired on a Helios™ (Standard BioTools). Acquired samples were randomized using Gaussian negative half zero randomization, normalized using bead normalization and concatenated in CyTOF Software version 6.7. Barcoded Flow Cytometry Standard (FCS) files were uploaded into OMIQ analysis software, where cell debris, beads and cell doublets were removed. Clean FCS files were then imported in R for debarcoding using CATALYST. Debarcoded FCS files were uploaded into OMIQ and live CD45^+^ cells were selected. Batch alignment and normalization were done based on the reference samples using CytoNorm in OMIQ (38). For clustering and immune population discovery, OMIQ was used. In detail, we first subsampled each FCS file to include 50.000 cells. Data was visualized using Uniform Manifold Approximation and Projection (UMAP) and cells were appointed to clusters by Phenotyping by Accelerated Refined Community (PARC) (39, 40). Visual inspection of the PARC-derived clusters and the clustered heatmaps that compare median marker expression between clusters allow us to manually merge clusters and to biologically annotate relevant immune cell subsets. Counts per immune cell subset were exported from OMIQ and their fractions of the total amount of immune cells per individual were calculated. Furthermore, we merged clusters as “parents” to provide easier depictions of the results (e.g. clusters CD14^+^ and CD16^+^ monocytes as parent cluster “monocytes”).

#### 2.3.2 Microbiota profile

Fecal and oral samples were collected in 1 mL eNat^®^ buffer (608CS01R, Copan) to determine the composition of the gut and oral microbiota. Samples were stored in -20°C until analysis. For fecal swabs, easyMAG lysis buffer was added to the swab in a 1:2 ratio and shaken at 1400 rounds per minute for 5 minutes at room temperature. One mL of the mixture was transferred to an Eppendorf tube and centrifuged (18,000 RCF; 2 minutes). Subsequently, 400 µL of the supernatant was transferred to an easyMAG isolation container containing 2 mL of easyMAG lysis buffer. For oral swabs, the same steps were performed, except that easyMAG lysis buffer was added to the swab in a 1:1 ratio, and 200 µL of the supernatant was used for DNA isolation. DNA was extracted with the NUCLISENS easyMAG kit (Biomérieux) according to the manufacturer’s instructions, using the “specific A” protocol, eluting DNA in 110 µL buffer.

The composition of the gut and oral microbiota was determined by using the interspace-profiling (IS-pro) technique, which is a clinically validated molecular assay for analysis of complex microbiota (41). The IS-pro technique (inBiome) identifies bacteria based on specific-length polymorphisms in the 16S-23S rDNA interspace (IS) region, combined with phylum-specific sequence polymorphisms in the 16S rDNA (41). Two polymerase chain reaction (PCR) reactions per DNA sample were performed according to the manufacturer’s instructions. In the first PCR reaction the IS-fragments of bacteria belonging to the phyla *Firmicutes, Bacteroidetes, Actinobacteria, Fusobacteria* and *Verrucomicrobia* were amplified, and in the second PCR reaction the IS fragments of the bacteria belonging to the phylum *Proteobacteria* and an internal amplification control were amplified. Fluorescent labeled IS-fragments were separated by length and detected on the ABI3500XL Genetic Analyzer (ThermoFisher). Data was preprocessed and species calling was done with the software suite antoni (Inbiome) and further analyzed with the Spotfire software package (TIBCO).

#### 2.3.3 Response status

For this pilot study, a patient was defined as a responder if there was no evidence of disease activity (NEDA), which is a composite outcome and consists of the occurrence of relapses, EDSS progression and radiological activity (NEDA-3) at the M12 visit (42). In case of missing data, the response status could still be determined if one of the available parameters classified the patient as a non-responder. In other cases, the response status was set as ‘missing’.

A relapse was defined as the development of new or worsening neurological symptoms attributable to MS. These symptoms must persist for more than 24 hours, should be in the absence of fever and must be preceded by a stable or improving neurological state for at least 30 days. The symptoms must be accompanied by objective neurological worsening consistent with an increase of at least one point in at least one functional system of the EDSS or an increase of 0.5 point on the EDSS. Changes in bowel and bladder or cerebral functions should not exclusively be responsible for documentation of a relapse.

EDSS progression was defined as a change in EDSS score from baseline to the M12 visit of at least 1.5 points if the baseline EDSS score was 0, one point if the baseline EDSS score was 1.0 to 5.0 and 0.5 point if the baseline EDSS score was 5.5 or more.

Radiological activity was defined as the development of new lesions, growth of existing lesions or gadolinium enhancement of new or existing lesions on brain MRI between the baseline MRI and the MRI that was performed around the M12 visit.

### 2.4 Statistical analysis

Baseline characteristics are presented as absolute and relative frequencies for categorical variables and median (interquartile range, IQR) for continuous data. The immunological subsets are displayed as median percentages of the total amount of immune cells. The microbiological measures are displayed as relative abundances. Transformation of the variables did not lead to a normal distribution.

Changes over time of immunological and microbiological measures were analyzed using linear mixed models (LMM), where time was used as fixed effect and patient as random intercept. Missing values were excluded pairwise. Bacterial species were analyzed if they were present in ≥25% of the samples. The assumption of a normal distribution of the residuals was met, except for the LMM of several bacterial species. These values were arcsine transformed, which led to a normal distribution of residuals in approximately 25% of the parameters. In cases where the final Hessian matrix was not positive definite, a different LMM was used without a random intercept for patient, but with an unstructured residual covariance matrix to model the dependency in the repeated measurements.

LMM were also used to analyze differences over time between responders and non-responders, adding response status as interaction term to the fixed effects. Differences in baseline characteristics between responders and non-responders were calculated using a t-test, Mann-Whitney U test or Fisher’s exact test where appropriate.

We checked if baseline characteristics were associated with baseline levels of the immunological subsets and microbiological measures using a Mann-Whitney U test, Kruskal Wallis test or Spearman’s Rho test for characteristics that were not normally distributed and a t-test, one-way ANOVA test or Pearson’s Rho test for characteristics that were normally distributed.

Statistical analyses were carried out with SPSS version 24. P-values were adjusted for multiple testing using the false discovery rate of Benjamini and Hochberg (43).

### 2.5 Ethics

This study was conducted in accordance with the Declaration of Helsinki. The study was approved by the medical ethics committee of Brabant, Tilburg, the Netherlands (NL66614.028.18). All patients provided written informed consent before entering the study.

### 2.6 Data availability

The raw CyTOF and IS-pro results can be found in online repositories upon publication of the manuscript (CyTOF: repository ID FR-FCM-Z8CL, http://flowrepository.org/id/RvFrNL592KHhSqfrRy8t4c3sm9MAUY0rFfnjL10lYDzveqjROZBbS1hMcW3JDaaK, and IS-pro: https://doi.org/10.17026/LS/GDHNO0). Corresponding metadata will be available from the corresponding author upon reasonable request.

## 3 Results

### 3.1 Baseline characteristics

A total of 25 patients were included in this analysis. Baseline characteristics are summarized in **Table 2**. The median age of the patients was 39 years (IQR 33-48 years), and 64% were female. The median disease duration was seven years (IQR 2.5-13.5 years), and the median EDSS at enrollment was 2.0 (IQR 2.0-3.0). A total of 18 patients (72%) started cladribine tablets because of suffering from disease activity, of whom 14 out of 18 patients have been using other IMT in the last six months. IMT used in the last six months consisted of platform therapies (glatiramer acetate, dimethylfumarate and teriflunomide) in 76% and of highly active therapies (fingolimod and ocrelizumab) in 24% of the patients. Two patients were treatment-naïve.

**Table 2:** Baseline characteristics.

Characteristics	All patients (N=25)
Age, years, median (IQR)	39 (33–48)
Female, n (%)	16 (64)
Body mass index, median (IQR)	25.4 (21.6-29.1)
Disease duration, years, median (IQR)	7 (2.5-13.5)
Documented relapses in past 2 years, median (IQR)	1 (0-1)
IMT used in last 6 months, n (%)	
Glatiramer acetate	5 (20)
Dimethylfumarate	7 (28)
Teriflunomide	4 (16)
Fingolimod	4 (16)
Ocrelizumab	1 (4)
None	4 (16)
Reason for switch to/start of cladribine tablets, n (%)	
Disease activity	18 (72)
Adverse events	7 (28)
Use of systemic corticosteroids in previous month, n (%)	1 (4)
Use of antibiotics in previous month, n (%)	2 (8)
Smoking, n (%)	
Yes	6 (24)
Past	10 (40)
Never	9 (36)
EDSS at enrollment, median (IQR)	2.0 (2.0-3.0)

IQR, interquartile range; IMT, immunomodulatory therapy; EDSS, expanded disability status scale.

### 3.2 Immunological profile

For the analysis of the immunological profile, samples were available at baseline and month 3 follow-up for all patients. For the M12 follow-up, samples were available for nineteen patients (**Supplementary Figure S1**). Six of these samples were obtained more than twelve months after the first dose of cladribine, but before the second treatment cycle.

**Figure 2:**
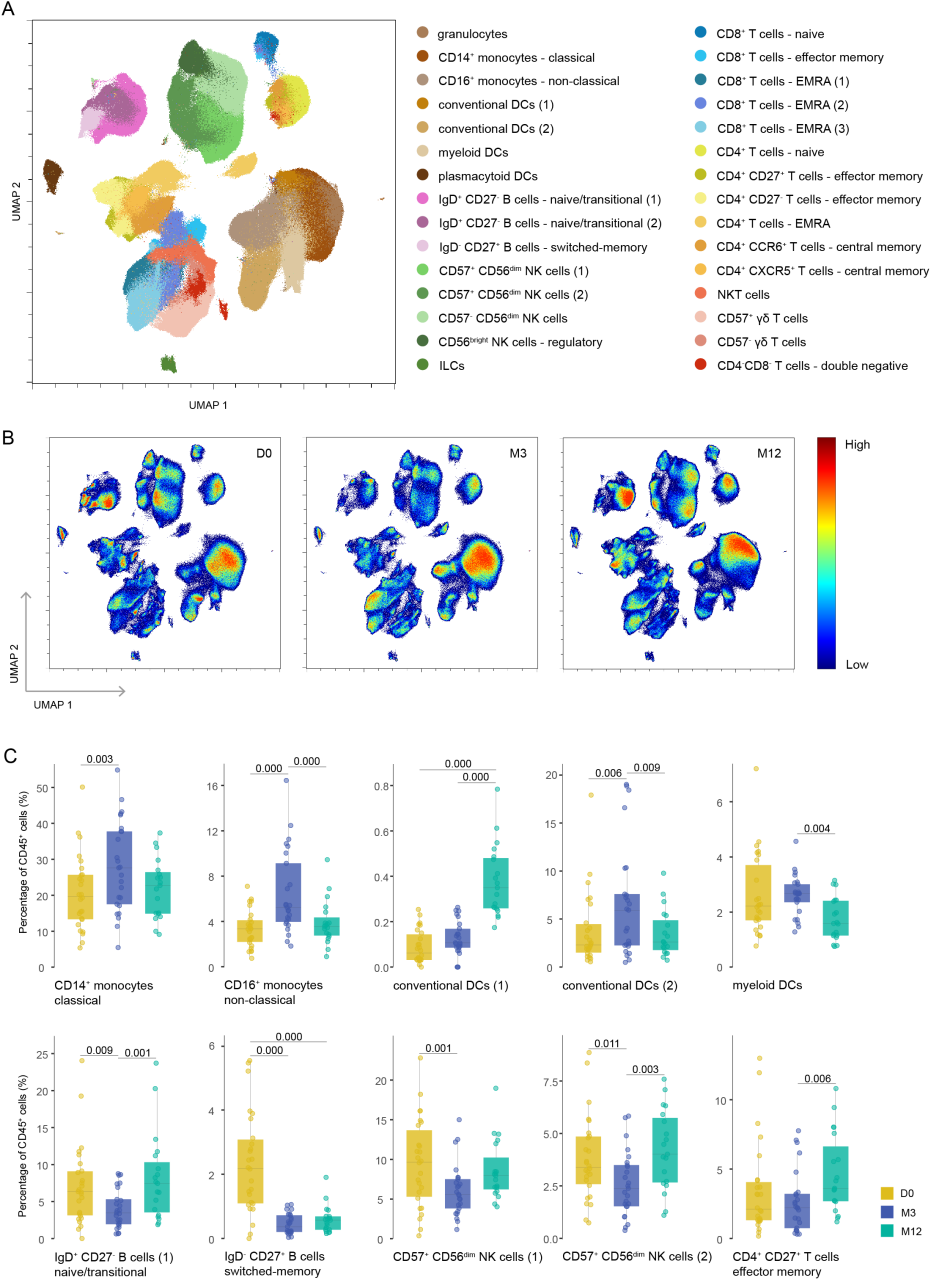
Different repopulation of various immune cells after cladribine treatment. **(A)** UMAP plot displaying CD45^+^ immune cells from the blood of cladribine-treated patients at different time points. Colors correspond to PARC-guided clustering. **(B)** Density UMAP plots showing the proportion of cells assigned to each time point according to each cluster. Color key indicates high (red) and low (blue) percentage of immune cells. **(C)** Percentage of each annotated cell population out of the total CD45^+^ immune cells at each time point. Each data point corresponds to each individual, colored by time point. Significant P-values are stated in the graphs. D0: baseline visit; M3: 3 month visit; M12: 12 month visit.

Using an unbiased approach, we identified 30 different clusters (**Figure 2A**, **Supplementary Figure S2A**), which we could manually merge into smaller parent clusters and seven known parent immune cell subsets, such as innate lymphocyte cells (ILCs, not including NK cells), granulocytes, monocytes, dendritic cells (DCs), B cells, NK cells and T cells (**Supplementary Table 1**).

We found a significant decline from baseline to M3 of B cells, specifically of naïve and transitional B cells (cluster IgD^+^ CD27^-^ B cells-1) and memory B cells (cluster IgD^-^ CD27^+^ B cells), and of CD57^+^ CD56^dim^ NK cells (clusters CD57^+^ CD56^dim^ NK cells-1 and -2) (**Figures 2B, C**). CD4^+^ T cells, more specifically CD4^+^ naïve T cells, and CD8^+^ naïve T cells showed a non-significant decline from baseline to M3 (**Supplementary Table 2**). In contrast, both clusters of CD14^+^ and CD16^+^ monocytes were relatively increased, as were both clusters of conventional dendritic cells and myeloid dendritic cells (**Figures 2B, C**). From M3 to M12, the values changed back to baseline levels (although not always statistically significant), except for the memory B cells. We also found a relative incline of T effector memory cells (cluster CD4^+^ CD27^+^ T effector memory cells) (**Figures 2B, C**). The manually merged clusters that were also significantly changed over time are shown in **Supplementary Figure S2B**. The baseline values were not correlated with any of the baseline characteristics.

### 3.3 Microbiological profile

**Figure 3:**
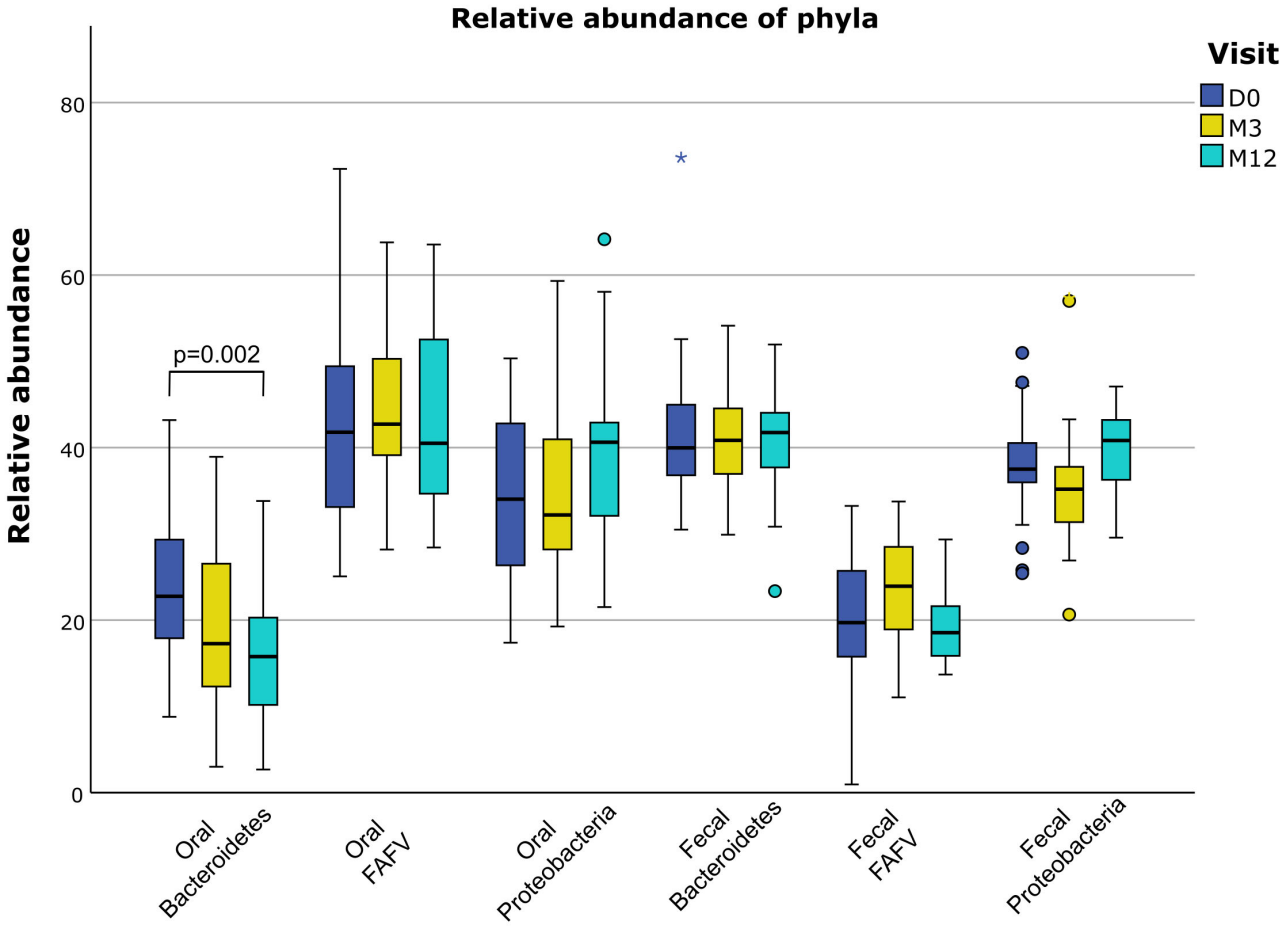
Relative abundance of phyla in oral and fecal samples. Significant P-values are stated in the graphs. FAFV: Firmicutes, Actinobacteria, Fusobacteria and Verrucomicrobia. D0: baseline visit; M3: 3 month visit; M12: 12 month visit. Dots represent outliers and asterixes represent extreme outliers.

For the analysis of the microbiological profile, samples were available at baseline and M3 follow-up for all patients. For the M12 follow-up, fecal samples were available for 20 patients and oral samples were available for 21 patients (**Supplementary Figure S1**). On a phylum-level, we found a significant decline from baseline to M12 for the relative abundance of the phylum *Bacteroidetes* in the oral samples (**Figure 3**). No significant changes were found in both oral and fecal samples from baseline to M3 for the three phylae, nor for alpha diversity and *Firmicutes/Bacteroidetes* ratio (**Supplementary Table S3**). Analysis on the species-level also did not show significant changes over time. The values we found at baseline were not correlated with baseline characteristics.

### 3.4 Responders versus non-responders

A total of 22 patients had sufficient data to determine the response status (**Supplementary Figure S1**). The total number of patients who were considered a responder at M12 was ten (40%). These patients had no relapses, EDSS progression and radiological activity. In the group of twelve non-responders, a relapse was present in two patients (17%), progression on the EDSS was present in five patients (42%) and radiological disease activity was present in seven patients (58%) (**Table 3**). Baseline characteristics were not significantly different between responders and non-responders (**Supplementary Table S4**).

**Table 3:** Response status.

Response status	Number of patients
Non-responder	12 (48%)
* Radiological disease activity^a^*	7 (58%)
* Relapse^a^*	2 (17%)
* Disability progression^a^*	5 (42%)
Responder	10 (40%)
Missing response status	3 (12%)^b^

a Percentages don’t add up to 100 because of overlap of categories (1 patient had a relapse and disability progression, 1 patient had a relapse and radiological disease activity).

b Two missing values were due to missing EDSS at M12 (and no radiological disease activity or relapse), one missing value was due to missing both EDSS and MRI at M12 (and no relapse). Two other patients were missing an MRI or EDSS, but their response status could be determined based on the parameters that were available (EDSS progression and radiological disease activity respectively).

**Figure 4:**
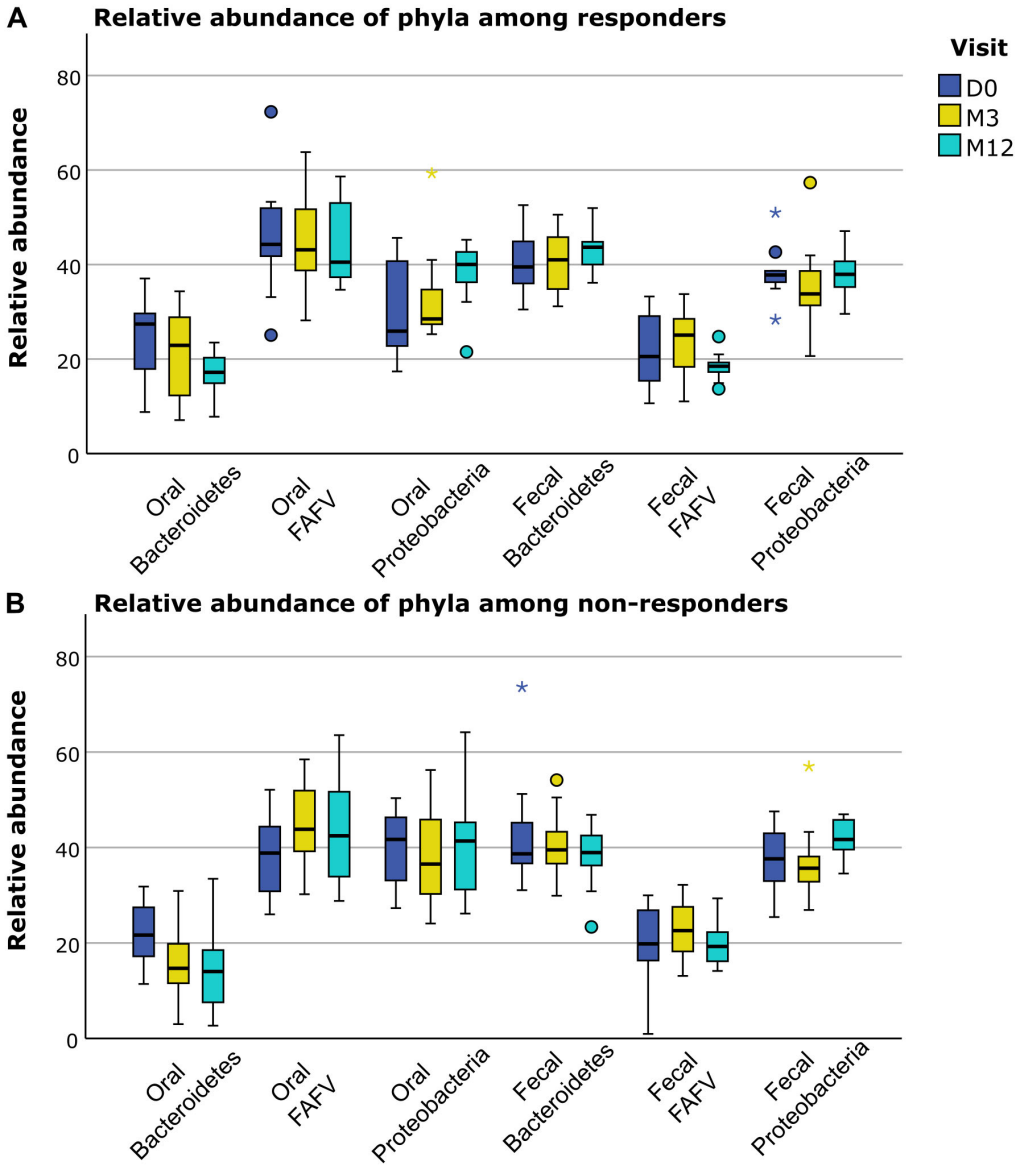
Differences in relative abundance of phyla between responders and non-responders. **(A)** Distribution of relative abundance of different phyla in fecal and oral samples in responders. **(B)** Distribution of relative abundance of different phyla in fecal and oral samples in non-responders. FAFV: Firmicutes, Actinobacteria, Fusobacteria and Verrucomicrobia. D0: baseline visit; M3: 3 month visit; M12: 12 month visit. Dots represent outliers and asterixes represent extreme outliers.

**Figure 5:**
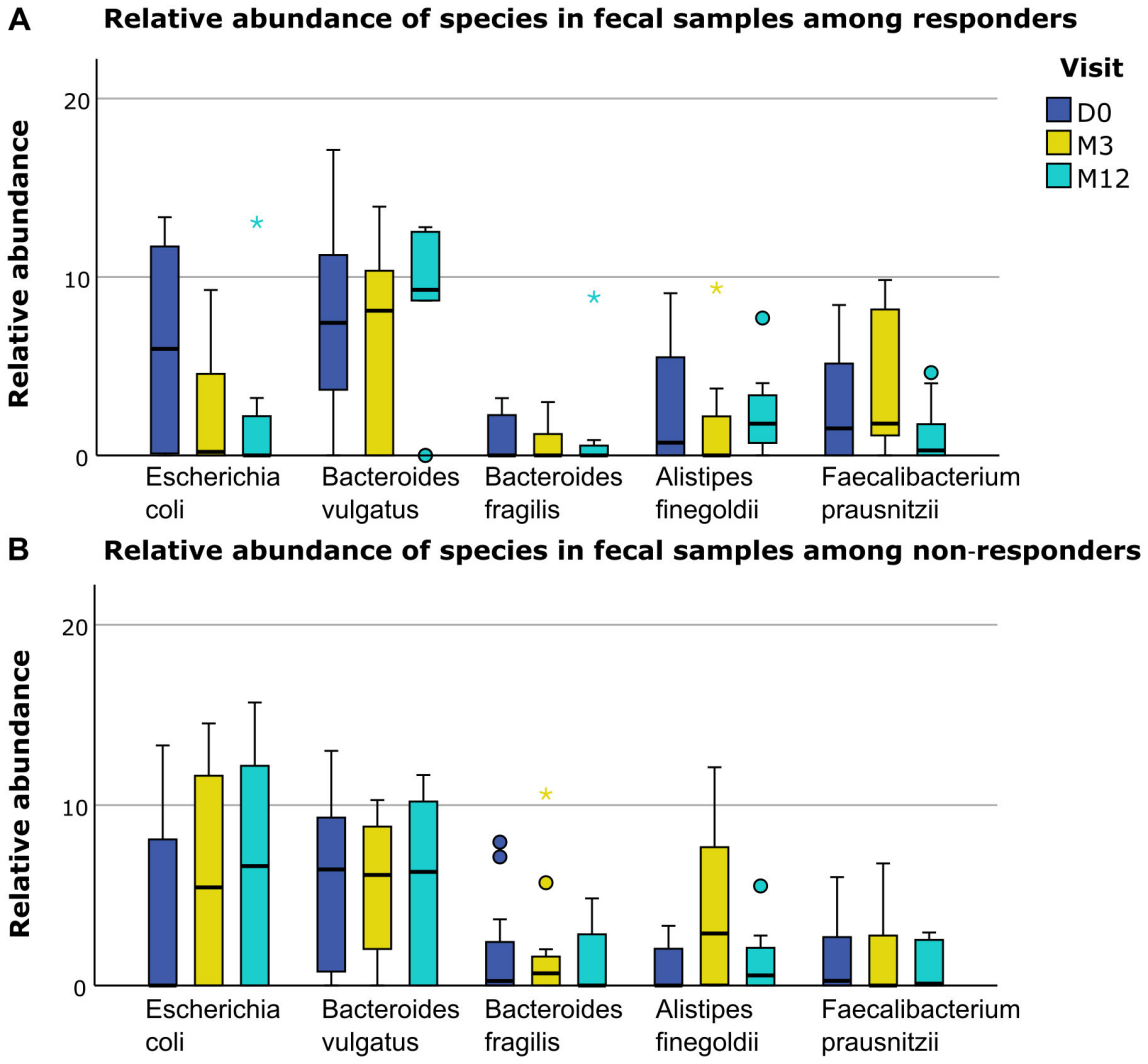
Differences in relative abundance of fecal species between responders and non-responders. **(A)** Relative abundance of several bacterial species in fecal samples in responders. **(B)** Relative abundance of several bacterial species in fecal samples in non-responders. D0: baseline visit; M3: 3 month visit; M12: 12 month visit. Dots represent outliers and asterixes represent extreme outliers.

**Figure 6:**
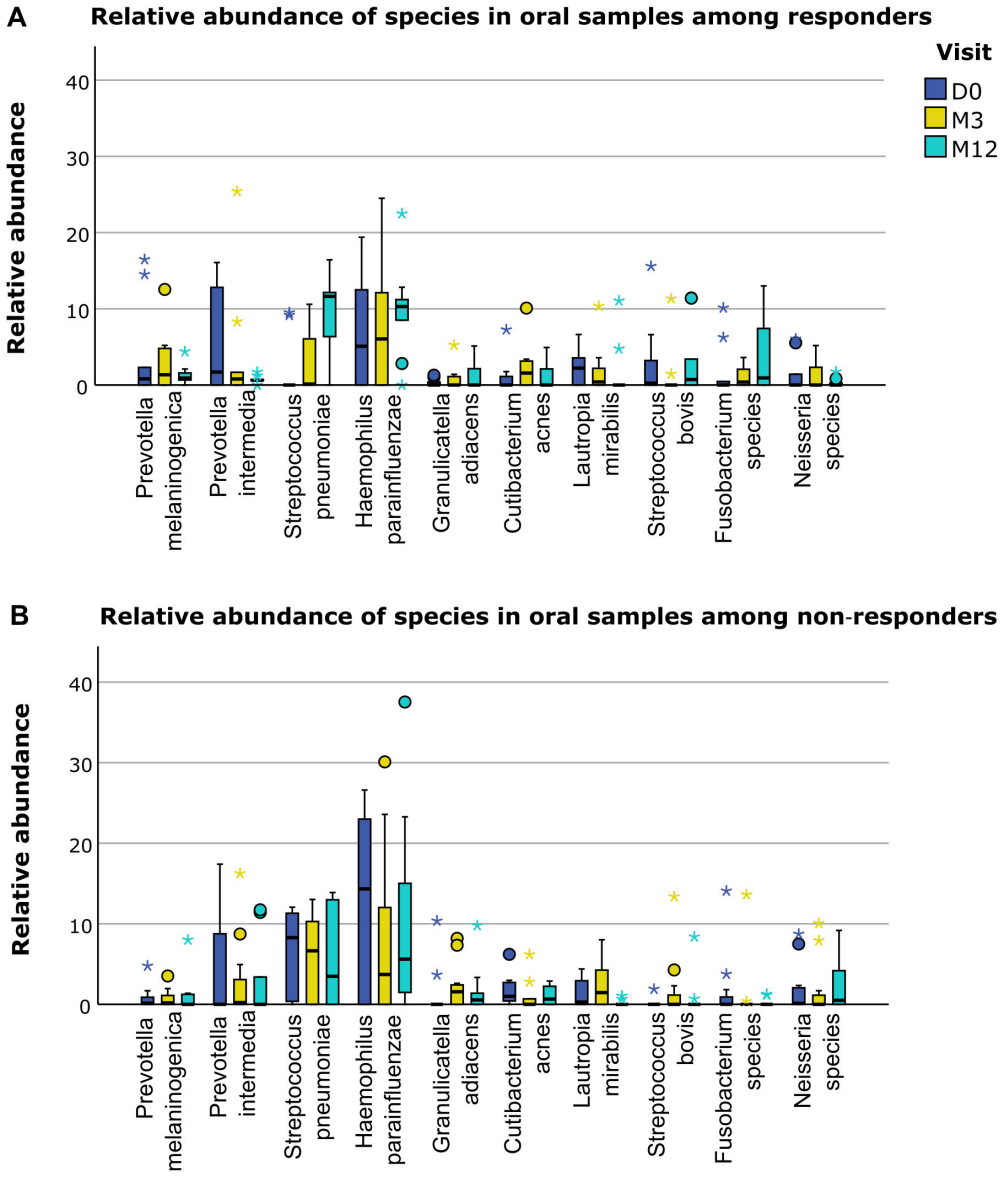
Differences in relative abundance of oral species between responders and non-responders. **(A)** Relative abundance of several bacterial species in oral samples in responders. **(B)** Relative abundance of several bacterial species in oral samples in non-responders. D0: baseline visit; M3: 3 month visit; M12: 12 month visit. Dots represent outliers and asterixes represent extreme outliers.

Baseline values and changes of immune profile and microbiological profile did not significantly differ between responders and non-responders (**Supplementary Table S5**). However, when we separately displayed the relative abundance of the different microbiological phylae and species for responders and non-responders, several trends for differences based on response status could be distinguished (**Figures 4**–**6**). For example, responders had a higher relative abundance of the phylum *Bacteroidetes* and a lower relative abundance of the phylum *Proteobacteria* compared to non-responders in their oral samples (*Bacteroidetes* at baseline, M3 and M12 in responders 27%, 23% and 17% and in non-responders 22%, 15% and 14%, and *Proteobacteria* at baseline, M3 and M12 in responders 26%, 28% and 40% and in non-responders 42%, 37% and 41%). In their oral samples at baseline, after three and after twelve months, responders had a higher relative abundance of two *Prevotella* species compared to non-responders (*Prevotella melaninogenica* 0.8%, 1.4% and 0.9% for responders versus 0.1%, 0.2% and 0% for non-responders and *Prevotella intermedia* 1.7%, 0.8% and 0.6% for responders versus 0%, 0.2% and 0% for non-responders). In their fecal samples, responders had a higher relative abundance of *Faecalibacterium prausnitzii* at baseline, M3 and M12 (1.5%, 1.8% and 0.3% for responders versus 0.3%, 0% and 0.1% for non-responders). Also, they had a higher relative abundance of *Escherichia coli* in their baseline fecal sample, while a lower relative abundance was found at M3 and M12 (6%, 0.2% and 0% for responders versus 0%, 5.4% and 6.0% for non-responders).

## 4 Discussion

The aim of this study was to describe the changes in gut and oral microbiota and immunological profile after treatment with cladribine tablets between responders and non-responders. Our data show that three months after treatment with cladribine tablets B cells and CD57^+^ CD56^dim^ NK cells significantly decline, and return to baseline levels twelve months after treatment, except for the memory B cells. A non-significant decline was found for CD4^+^ and CD8^+^ naïve T cells. Microbiological analysis shows a significant decline of the phylum *Bacteroidetes* in oral samples twelve months after treatment. No significant changes were found at the species level of both fecal and oral samples. We did not find significant differences for immunological and microbiological outcomes between responders and non-responders. However, the microbiological profile showed several differences in microbes with known anti- or pro-inflammatory properties between responders and non-responders.

Our immunological findings are in line with other studies investigating changes of immune subsets after treatment with cladribine (4–7, 9). B cells show significant alterations in MS patients compared to healthy controls, and treatment with B cell depleting therapies can successfully attenuate disease activity (9, 44, 45). The specific impact on memory B cells plays an important role in the long-lasting efficacy of immune reconstitution therapies (9). NK cells are part of the innate immune system and play an important role in the immune surveillance mechanisms for infections and cancer (8). Although NK cells significantly declined after cladribine treatment, the innate immune system function seems to remain relatively intact, as the rate of serious infectious adverse events are limited and no elevated risk of malignancies was reported (1, 3).

In other studies conflicting results were found on the correlation between response status and changes in the immunological system after treatment with cladribine tablets (7, 8). In comparison with other studies, here we have defined the immune landscape after treatment with cladribine tables using single cell mass cytometry, using CyTOF technology, instead of regular flowcytometry. This technique allowed us to identify a large number of different immune cell subsets in an unbiased manner and consequently, to understand the main immune cell subsets as well as the more rare cell subsets and their responses to cladribine tables. Hence, with this technique we can confirm the previous results regarding the main cell subsets and expand their findings to other immune cell types (4–8).

Our microbiological findings add new findings to the research field, as we did not find any research on changes of the microbiota after treatment with cladribine. However, several longitudinal studies investigated the changes of the microbiota after treatment with other IMT, like dimethylfumarate and ocrelizumab (21, 28–30). Also, several cross-sectional studies investigated differences between untreated and treated patients (18–20, 25, 27). These studies all were able to show differences after treatment (starting from one month after start of treatment), varying from differences on the phylum, genus or species level. However, there is little consistency in results, most likely caused by different study populations, treatment and sequencing strategies. Yet, we can find some overlap in the mechanisms by which these bacteria exert an immunomodulatory effect and accordingly why change of their abundance after treatment is possibly related to attenuated disease activity. The most consistently reported mechanism is the anti-inflammatory response of short-chain fatty acid producers, mostly of the phylum *Firmicutes*, but also some genera from the *Bacteroidetes* phylum (46–49). Another potential anti-inflammatory mechanism is the expansion of regulatory T cells by binding of Lipid 654 to toll-like receptor 2. Lipid 654 is a bacterially derived product from species from the *Bacteroidetes* phylum (46, 50). A third potential mechanism is the production of tryptophan by, among others, *Corynebacterium* species, *Streptococcus* species and *Escherichia coli*, which lead to a higher level of serotonin and subsequently reduction of anti-inflammatory cytokines (51–53). Lastly, an anti-inflammatory effect is seen in bacteria which play a role in the phytoestrogen metabolism, such as *Prevotella* and *Adlercreutzia (* 47, 54). Our finding that the phylum *Bacteroidetes* in oral samples declines twelve months after treatment is remarkable, as, based on previous literature and immunological findings, we expected to find changes already after three months. Also, we expected to find an incline instead of decline of the phylum *Bacteroidetes* as the genera and species belonging to this phylum possess mostly anti-inflammatory properties (46, 47, 51).

In our study, we showed that several phyla, genera or species with anti-inflammatory properties had a higher relative abundance in responders compared to non-responders (*Bacteroidetes* and *Prevotella* in oral samples, and *Faecalibacterium prausnitzii* in fecal samples), and the phyla *Proteobacteria* with pro-inflammatory properties had a lower relative abundance in responders compared to non-responders in oral samples, and a potentially pro-inflammatory species (*Escherichia coli*) showed a decrease after treatment in responders in fecal samples, which potentially explains the different treatment response (46, 47, 51). Several other studies were able to correlate microbiological changes to disease course (relapse risk, radiological disease activity), but data on the correlation of microbiological changes and response to treatment are scarce (26, 55, 56). Although species-level analysis provides a detailed understanding of the microbiological niche, due to the significant inter-patient diversity in the microbiota, species-level analysis alone can be limited in its interpretability and ability to capture broader patterns. Therefore, we decided to analyze the microbiota both on phylum-level and species-level, to provide a necessary overarching view of microbiota composition and trends that are less apparent at the species level. We acknowledge that filtering species that were present less than 25% of the samples may have excluded some species that could be relevant to the responder versus non-responder analysis, particularly rare species that might still hold clinical importance. However, this threshold was chosen to reduce noise and focus on more consistently observed species, given the challenges of analyzing microbiota data with a relatively small cohort. In a larger cohort, it might be possible to allow less stringent filtering thresholds and explore rare but potentially important species. Also, inherent to the IS-pro technique, it is possible that very low amounts of highly prevalent species with a low relative abundance, such as *Escherichia coli*, may have gone undetected in some participants. We consider these quantities unlikely to yield clinically relevant insights. Our current analysis focuses on broader patterns and trends in the microbiota, which we believe are adequately captured with the methodologies employed. We acknowledge our exploratory approach and its limitations, but would like to emphasize the strength of the longitudinal approach.

This study has several limitations. First, for this pilot study the sample size was small. Therefore, we were not able to correct for potential confounders, such as previous use of IMT, concomitant medication, diet and stool consistency. However, because of the longitudinal design of the study, where every patient act as its own control, the influence of these confounders are limited to a minimum. In the BIA study, eventually 81 patients are included. Therefore, in the future we will be able to expand our analysis to a larger cohort and correct for potential confounders. Second, the duration of the follow-up period of this study was only one year. We are collecting data to determine a response status two years after treatment, and we are planning an extension of the follow-up period until four years after start of treatment. Lastly, the response status was biased by the missing of a re-baseline MRI and by a possible shift to more non-responders due to missing data. In case of missing one out of three parameters to determine treatment response (relapse, EDSS and MRI) a patient cannot be defined as a responder, while only one positive parameter (a relapse, MRI with disease activity or progression on the EDSS) can make a patient a non-responder.

In future research, our analysis should be expanded to a larger sample size and longer follow-up, to confirm or reject our findings. At the moment, we are completing our database of 81 patients with a follow-up of two years after start of treatment with cladribine. The analysis we have done for the current study can then be expanded to a larger sample size. Also, we can research the association between changes in the immune system, the microbiota and response status.

In conclusion, after treatment with cladribine tablets, significant changes in the composition of the immune system are found. Also, the microbiological profile showed several promising differences between responders and non-responders. Future research on data from the BIA study, with a larger sample size and extended follow-up, can possibly confirm the reliability of our findings and expand our knowledge on the interplay between the immune system and the microbiota.

## Data Availability

The datasets presented in this study can be found in online repositories. The names of the repository/repositories and accession number(s) can be found below: http://flowrepository.org/id/RvFrNL592KHhSqfrRy8t4c3sm9MAUY0rFfnjL10lYDzveqjROZBbS1hMcW3JDaaK, Flow Repository, FR-FCM-Z8CL, available for reviewers via link, publicly available upon publication of the manuscript https://doi.org/10.17026/LS/GDHNO0, DANS Data Station Life Sciences.

## Collaborators

The BIA study group includes the following investigators: J de Beukelaar, MD, PhD, Neurologist, Department of Neurology, Albert Schweitzer Hospital, Dordrecht, the Netherlands; ML Boon, MSc, inBiome, Amsterdam, Netherlands; AE Budding, MD, PhD, inBiome, Amsterdam, Netherlands; J Fermont, MD, Neurologist, Department of Neurology, Amphia Hospital, Breda, the Netherlands; STFM Frequin, MD, PhD, Neurologist, Department of Neurology, St Antonius Hospital, Nieuwegein, the Netherlands; OHH Gerlach, MD, PhD, Neurologist, Department of Neurology, Zuyderland Medical Center, Sittard-Geleen, the Netherlands, and School for Mental Health and Neuroscience, Maastricht University, Maastricht, The Netherlands; J Killestein, Md, PhD, Neurologist, MS Center Amsterdam, Department of Neurology, Amsterdam Neuroscience, Amsterdam UMC Location Vrije Universiteit Amsterdam, Amsterdam, The Netherlands; J van Pamelen, MD, PhD, Resident Neurology, Department of Neurology, Elisabeth-TweeSteden Hospital, Tilburg, the Netherlands; L van Olst, MSc, PhD, Department of Molecular Cell Biology and Immunology, MS Center Amsterdam, Amsterdam Neuroscience, Amsterdam UMC Location Vrije Universiteit Amsterdam, Amsterdam, The Netherlands; BW van Oosten, MD, PhD, Neurologist, MS Center Amsterdam, Department of Neurology, Amsterdam Neuroscience, Amsterdam UMC Location Vrije Universiteit Amsterdam, Amsterdam, The Netherlands; SMA van der Pol, BsC, Department of Molecular Cell Biology and Immunology, MS Center Amsterdam, Amsterdam Neuroscience, Amsterdam UMC Location Vrije Universiteit Amsterdam, Amsterdam, The Netherlands; C Rodriguez-Mogeda, MSc, PhD, Department of Molecular Cell Biology and Immunology, MS Center Amsterdam, Amsterdam Neuroscience, Amsterdam UMC Location Vrije Universiteit Amsterdam, Amsterdam, The Netherlands; LH Visser, MD, PhD, Neurologist, Department of Neurology, Elisabeth-TweeSteden Hospital, Tilburg, the Netherlands; J van Vliet, MD, PhD, Neurologist, Department of Neurology, Jeroen Bosch Hospital, ‘s-Hertogenbosch, the Netherlands; and HE de Vries, MSc, PhD, Department of Molecular Cell Biology and Immunology, MS Center Amsterdam, Amsterdam Neuroscience, Amsterdam UMC Location Vrije Universiteit Amsterdam, Amsterdam, The Netherlands.

## Supplementary material

Supplementary Figure 1 Flowchart of included patients.

Supplementary Figure 2 Immune cell subclustering. **(A)** Heatmap displays the median scaled intensities of all the markers across the annotated immune cell clusters. Color key indicates high (red) and low (blue) median expression. **(B)** Percentage of parental immune cell populations out of the total CD45^+^ immune cells at each time point. Each data point corresponds to each individual, colored by time point. P-values are stated in the graphs. D0, day 0; M3, 3 months; M12, 12 months.
