# Supplementary figures and images for: The gut-brain-axis one year after treatment with cladribine tablets in patients with relapsing remitting multiple sclerosis: a pilot study
Source: Front Immunol. 2025 Feb 27;16:1514762. doi: 10.3389/fimmu.2025.1514762 (PMC11903281; doi:10.3389/fimmu.2025.1514762)

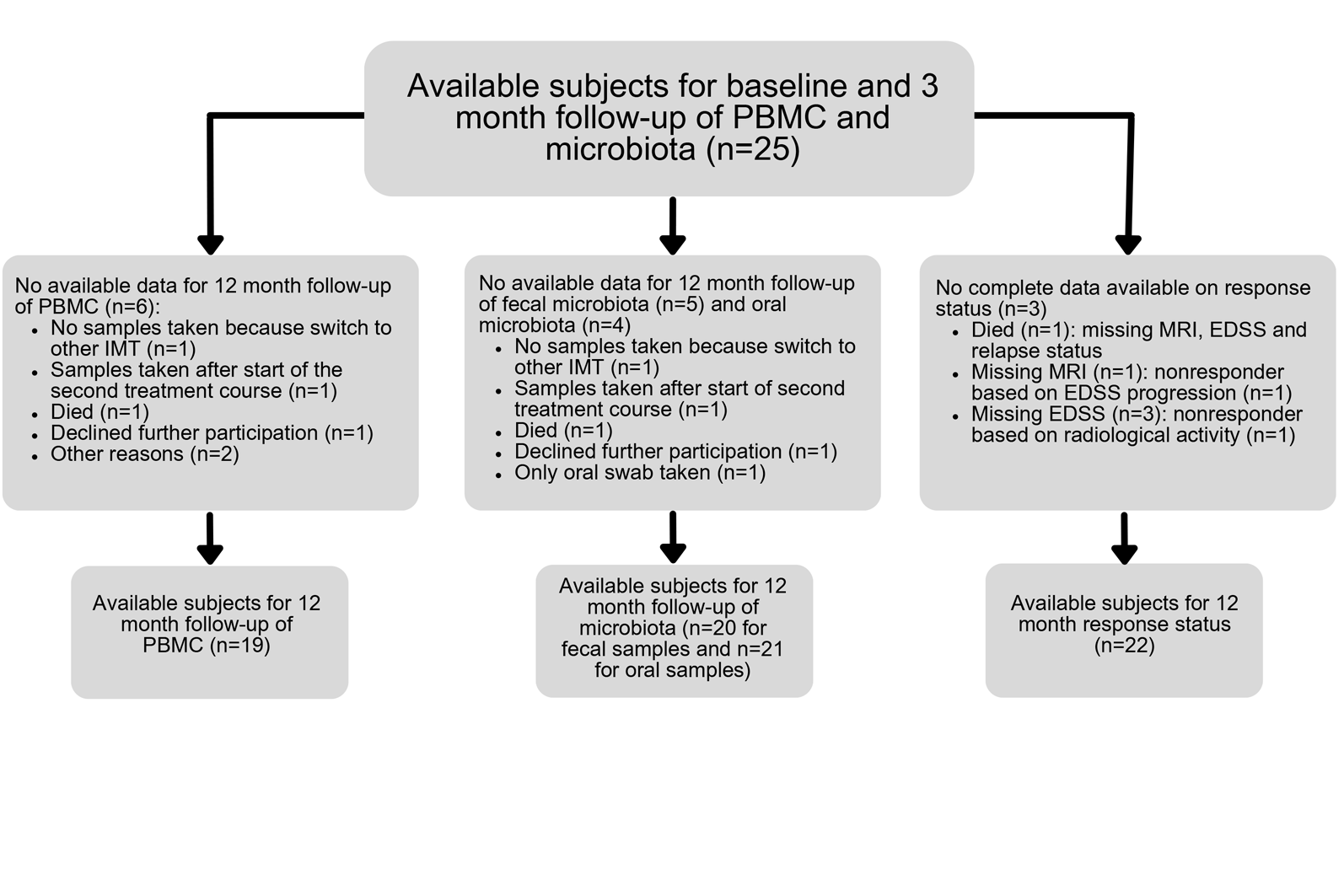

Supplement: Supplementary Figure 1 — Flowchart of included patients. [file Image1.tif]

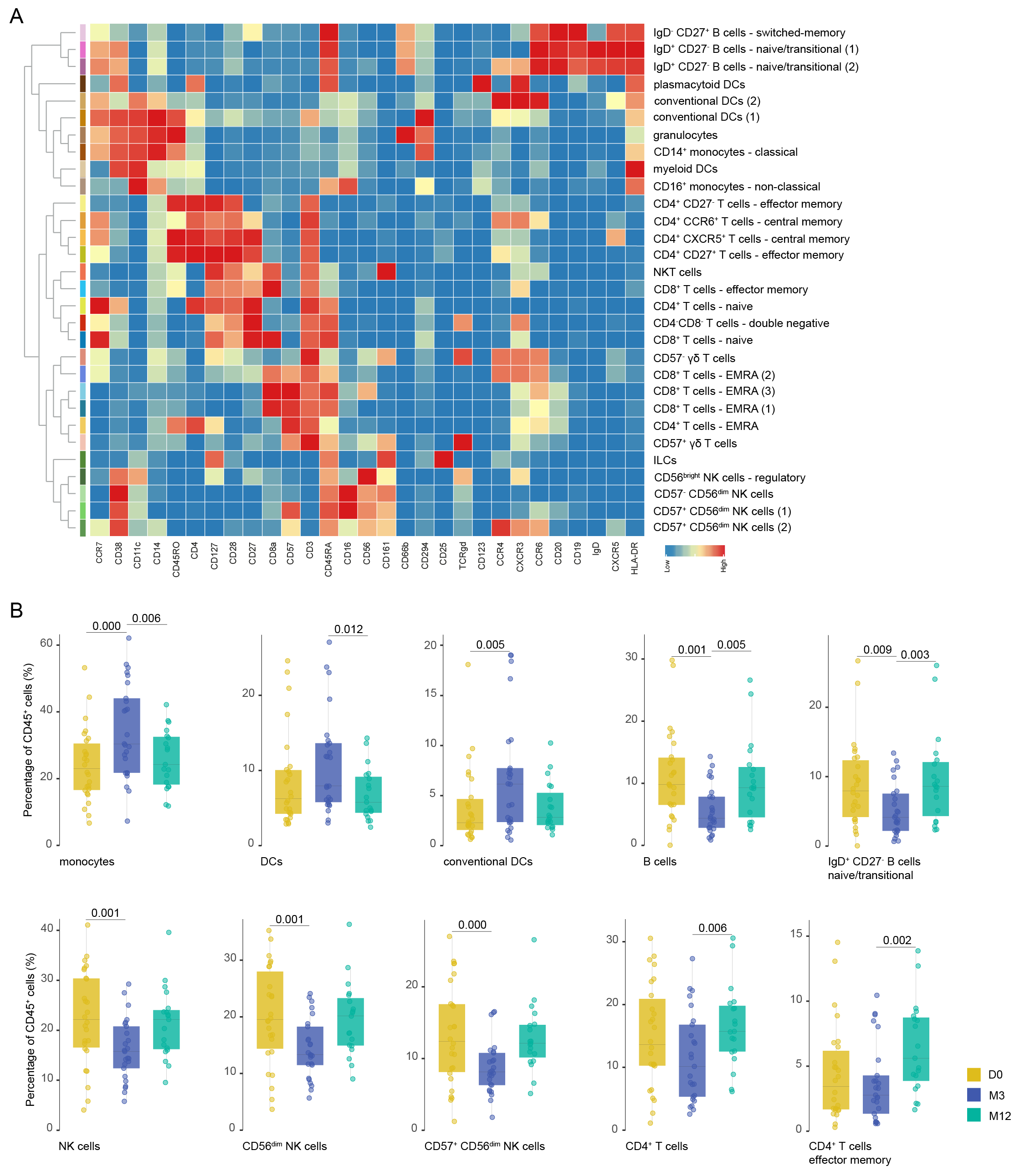

Supplement: Supplementary Figure 2 — Immune cell subclustering. (A) Heatmap displays the median scaled intensities of all the markers across the annotated immune cell clusters. Color key indicates high (red) and low (blue) median expression. (B) Percentage of parental immune cell populations out of the total CD45+ immune cells at each time point. Each data point corresponds to each individual, colored by time point. P-values are stated in the graphs. D0, day 0; M3, 3 months; M12, 12 months. [file Image2.tif]
