# Supplementary material for: The gut-brain-axis one year after treatment with cladribine tablets in patients with relapsing remitting multiple sclerosis: a pilot study
Source: Front Immunol. 2025 Feb 27;16:1514762. doi: 10.3389/fimmu.2025.1514762 (PMC11903281; doi:10.3389/fimmu.2025.1514762)
Supplement: Supplementary file 3 [file Table1.docx]

*Supplementary Table 1 Merging PBMC clusters.*

| **clusters** | **naming** | **merging step 1** | **merging step 2** | **final parent** |
| --- | --- | --- | --- | --- |
| 1 | granulocytes |  |  | granulocytes |
| 2 | CD14⁺ monocytes - classical | monocytes | | |
| 3 | CD16⁺ monocytes - non-classical |  |  |  |
| 4 | conventional DCs (1) | conventional DCs | | DCs |
| 5 | conventional DCs (2) |  |  |  |
| 6 | myeloid DCs |  |  |  |
| 7 | plasmacytoid DCs |  |  |  |
| 8 | IgD⁺ CD27⁻ B cells - naive/transitional (1) | naive/transitional | | B cells |
| 9 | IgD⁺ CD27⁻ B cells - naive/transitional (2) |  |  |  |
| 10 | IgD⁻ CD27⁺ B cells - switched-memory | switched memory | |  |
| 11 | CD57⁺ CD56^dim^ NK cells (1) | CD57⁺ CD56^dim^ NK cells | CD56^dim^ NK cells | NK cells |
| 12 | CD57⁺ CD56^dim^ NK cells (2) |  |  |  |
| 13 | CD57⁻ CD56^dim^ NK cells |  |  |  |
| 14 | CD56^bright^ NK cells - regulatory |  | CD56^bright^ NK cells |  |
| 15 | ILCs |  |  | ILCs |
| 16 | CD8⁺ T cells - naive |  | CD8⁺ T cells | T cells |
| 17 | CD8⁺ T cells - effector memory |  |  |  |
| 18 | CD8⁺ T cells - EMRA (1) | Temra |  |  |
| 19 | CD8⁺ T cells - EMRA (2) |  |  |  |
| 20 | CD8⁺ T cells - EMRA (3) |  |  |  |
| 21 | CD4⁺ T cells - naive |  | CD4⁺ T cells |  |
| 22 | CD4⁺ CD27⁺ T cells - effector memory | effector memory |  |  |
| 23 | CD4⁺ CD27⁻ T cells - effector memory |  |  |  |
| 24 | CD4⁺ T cells - EMRA |  |  |  |
| 25 | CD4⁺ CCR6⁺ T cells - central memory | central memory |  |  |
| 26 | CD4⁺ CXCR5⁺ T cells - central memory |  |  |  |
| 27 | NKT cells |  |  |  |
| 28 | CD57⁺ γδ T cells | γδ T cells | |  |
| 29 | CD57⁻ γδ T cells |  |  |  |
| 30 | CD4⁻ CD8⁻ T cells - double negative |  |  |  |
