# Supplementary material for: The gut-brain-axis one year after treatment with cladribine tablets in patients with relapsing remitting multiple sclerosis: a pilot study
Source: Front Immunol. 2025 Feb 27;16:1514762. doi: 10.3389/fimmu.2025.1514762 (PMC11903281; doi:10.3389/fimmu.2025.1514762)
Supplement: Supplementary file 4 [file Table2.docx]

| **Subsets** | **D0 (median)** | **M3 (median)** | **M12 (median)** | **β (SE)**  **D0-M3** | **P-value D0-M3** | **β (SE)**  **D0-M12** | **P-value D0-M12** | **β (SE)**  **M3-M12** | **P-value M3-M12** |
| --- | --- | --- | --- | --- | --- | --- | --- | --- | --- |
| ILC | 0,28 | 0,20 | 0,16 | -0,05 (0,05) | 0,342 | -0.09 (0,05) | 0,086 | -0,05 (0,05) | 0,382 |
| GRAN | 0,07 | 0,10 | 0,18 | 0,02 (0,07) | 0,780 | 0,04 (0,07) | 0,591 | 0,02 (0,07) | 0,777 |
| MONO_TOT | 21,89 | 30,42 | 24,25 | 10,74 (2,50) | **0,000** | 2,89 (2,75) | 0,298 | -7,85 (2,75) | **0,006** |
| CD14_mono | 19,30 | 27,63 | 22,72 | 7,54 (2,36) | **0,003** | 2,21 (2,59) | 0,399 | -5,34 (2,59) | 0,045 |
| CD16_mono | 3,32 | 5,22 | 3,58 | 3,20 (0,52) | **0,000** | 0,65 (0,57) | 0,261 | -2,55 (0,57) | **0,000** |
| DC_TOT | 5,97 | 7,93 | 5,80 | 2,34 (1,26) | 0,071 | -1,30 (1,39) | 0,353 | -3,64 (1,39) | **0,012** |
| cDC_TOT | 2,23 | 6,14 | 2,85 | 2,99 (1,01) | **0,005** | 0,23 (1,11) | 0,833 | -2,76 (1,11) | 0,016 |
| cDC_1 | 0,05 | 0,11 | 0,35 | 0,04 (0,02) | 0,152 | 0,30 (0,03) | **0,000** | 0,26 (0,03) | **0,000** |
| cDC_2 | 2,20 | 5,93 | 2,59 | 2,95 (1,01) | **0,006** | -0,07 (1,11) | 0,953 | -3,02 (1,11) | **0,009** |
| mDCs | 2,24 | 2,67 | 1,59 | -0,01 (0,23) | 0,953 | -0,77 (0,25) | 0,004 | -0,76 (0,25) | **0,004** |
| pDCs | 1,23 | 1,14 | 0,84 | -0,64 (0,49) | 0,199 | -0,83 (0,53) | 0,126 | -0,19 (0,53) | 0,725 |
| B_TOT | 9,77 | 4,36 | 9,25 | -5,39 (1,44) | **0,001** | -0,80 (1,57) | 0,615 | 4,60 (1,57) | **0,005** |
| B_CD27NEG_TOT | 7,70 | 4,16 | 8,62 | -3,54 (1,29) | **0,009** | 0,91 (1,41) | 0,523 | 4,45 (1,41) | **0,003** |
| B_CD27NEG_1 | 6,30 | 3,48 | 7,45 | -3,04 (1,11) | **0,009** | 1,10 (1,22) | 0,370 | 4,13 (1,22) | **0,001** |
| B_CD27NEG_2 | 1,29 | 0,68 | 1,18 | -0,50 (0,27) | 0,072 | -0,17 (0,30) | 0,569 | 0,33 (0,30) | 0,274 |
| B_CD27POS | 2,16 | 0,37 | 0,57 | -1,85 (0,27) | **0,000** | -1,71 (0,29) | **0,000** | 0,15 (0,29) | 0,617 |
| NK_TOT | 21,58 | 15,74 | 22,15 | -6,23 (1,66) | **0,001** | -2,42 (1,82) | 0,190 | 3,80 (1,82) | 0,043^c^ |
| NK56_DIM_TOT | 19,31 | 13,31 | 20,13 | -5,74 (1,63) | **0,001** | -1,55 (1,79) | 0,390 | 4,19 (1,79) | 0,023 |
| NK56_DIM_57POS_TOT | 12,72 | 8,12 | 12,12 | -4,67 (1,23) | **0,000** | -1,33 (1,34) | 0,328 | 3,35 (1,34) | 0,017 |
| NK56_DIM_57POS_1 | 9,78 | 5,59 | 7,93 | -3,44 (0,96) | **0,001** | -1,66 (1,06) | 0,122 | 1,78 (1,06) | 0,100 |
| NK56_DIM_57POS_2 | 3,44 | 2,38 | 4,01 | -1,23 (0,47) | **0,011** | 0,33 (0,51) | 0,515 | 1,56 (0,51) | **0,003** |
| NK56_DIM_57NEG | 6,70 | 5,38 | 5,65 | -1,07 (0,56) | 0,065 | -0,39 (0,62) | 0,531 | 0,68 (0,62) | 0,284 |
| NK56_BR | 2,11 | 1,01 | 1,46 | -0,49 (0,25) | 0,063 | -0,71 (0,28) | 0,016 | -0,22 (0,28) | 0,442 |
| T_TOT | 32,95 | 33,56 | 33,98 | -1,43 (2,59) | 0,583 | 1,60 (2,84) | 0,577 | 3,03 (2,84) | 0,292 |
| NK_T_TOT | 0,25 | 0,34 | 0,80 | 0,20 (0,23) | 0,375 | 0,33 (0,25) | 0,187 | 0,13 (0,25) | 0,604 |
| GD_T_TOT | 2,38 | 3,61 | 3,53 | 1,21 (0,50) | 0,019 | 0,49 (0,55) | 0,378 | 0,72 (0,55) | 0,201 |
| GD_T_57POS | 1,85 | 3,14 | 2,92 | 1,05 (0,43) | 0,019 | 0,24 (0,48) | 0,620 | -0,81 (0,48) | 0,095 |
| GD_T_57NEG | 0,49 | 0,69 | 0,76 | 0,15 (0,12) | 0,200 | 0,23 (0,13) | 0,079 | 0,08 (0,13) | 0,534 |
| CD4_TOT | 14,11 | 10,14 | 15,72 | -3,55 (1,47) | 0,020 | 1,12 (1,62) | 0,491 | 4,67 (1,62) | **0,006** |
| CD4_TCM_TOT^a^ | 2,61 | 2,08 | 2,27 | -0,81 (0,68) | 0,242 | -0,24 (0,66) | 0,727 | 0,58 (0,68) | 0,403 |
| CD4_TCM_CCR6POS | 2,28 | 1,64 | 1,56 | -0,58 (0,66) | 0,384 | -0,33 (0,71) | 0,646 | 0,25 (0,71) | 0,724 |
| CD4_TCM_CXCR5POS | 0,40 | 0,29 | 0,59 | -0,23 (0,10) | 0,024 | -0,01 (0,11) | 0,898 | 0,21 (0,11) | 0,051 |
| CD4_NAIVE | 3,73 | 1,36 | 3,97 | -2,06 (0,93) | 0,032 | -1,14 (1,02) | 0,269 | 0,92 (1,02) | 0,370 |
| CD4_TEMRA | 0,26 | 0,23 | 0,59 | 0,07 (0,54) | 0,903 | 0,42 (0,60) | 0,482 | 0,36 (0,60) | 0,553 |
| CD4_TEM_TOT | 2,98 | 2,78 | 5,61 | -0,74 (0,76) | 0,334 | 1,94 (0,83) | 0,024 | 2,69 (0,83) | **0,002** |
| CD4_TEM_CD27POS | 2,02 | 2,21 | 3,62 | -0,80 (0,65) | 0,225 | 1,25 (0,72) | 0,087 | 2,05 (0,72) | **0,006** |
| CD4_TEM_CD27NEG | 0,59 | 0,57 | 1,42 | 0,06 (0,23) | 0,792 | 0,68 (0,25) | 0,010 | 0,62 (0,25) | 0,018 |
| CD8_TOT | 10,62 | 10,32 | 12,28 | 0,70 (1,47) | 0,638 | -0,08 (1,62) | 0,959 | -0,78 (1,62) | 0,632 |
| CD8_TEM | 2,37 | 2,04 | 2,55 | 0,30 (0,53) | 0,571 | 0,55 (0,59) | 0,353 | 0,25 (0,59) | 0,675 |
| CD8_NAIVE_TOT | 1,08 | 0,36 | 1,25 | -0,47 (0,28) | 0,103 | -0,22 (0,31) | 0,474 | 0,25 (0,31) | 0,429 |
| CD8_TEMRA_TOT | 7,03 | 8,18 | 8,75 | 0,87 (1,24) | 0,487 | -0,38 (1,36) | 0,783 | -1,24 (1,36) | 0,366 |
| CD8_TEMRA_1 | 3,45 | 3,34 | 3,21 | 0,69 (0,78) | 0,382 | -0,99 (0,86) | 0,257 | -1,69 (0,86) | 0,057 |
| CD8_TEMRA_2 | 1,83 | 1,73 | 2,52 | -0,35 (0,30) | 0,257 | 0,20 (0,33) | 0,544 | 0,55 (0,33) | 0,103 |
| CD8_TEMRA_3 | 0,73 | 1,22 | 1,66 | 0,52 (0,41) | 0,212 | 0,33 (0,46) | 0,481 | -0,20 (0,46) | 0,668 |
| DN_T_TOT | 0,22 | 0,20 | 0,24 | 0,01 (0,03) | 0,795 | 0,03 (0,04) | 0,362 | 0,03 (0,04) | 0,498 |

*Median values represent % of total immunological cells. P-values, β and SE (standard error) represent results of the linear mixed model analysis.
Bold type indicates statistical significance after correction for multiple testing.
a Final Hessian matrix not positive definite (different model used)*

*Abbreviations: D0 = baseline, M3 and M12 = follow-up after 3 and 12 months. ILC = innate lymphoid cells, GRAN = granulocytes, MONO = monocytes, TOT = total, cDC = conventional dendritic cells, mDC = myeloid dendritic cells, pDC = plasmacytoid dendritic cells, NEG = negative, POS = positive, NK = natural killer, BR = bright, NK_T = natural killer T cell, GD_T = γδ T cell, TCM = central memory T cell, TEMRA = effector memory cell re-expressing CD45RA, TEM = effector memory , DN_T = double negative T cell.*
