# Supplementary material for: The gut-brain-axis one year after treatment with cladribine tablets in patients with relapsing remitting multiple sclerosis: a pilot study
Source: Front Immunol. 2025 Feb 27;16:1514762. doi: 10.3389/fimmu.2025.1514762 (PMC11903281; doi:10.3389/fimmu.2025.1514762)
Supplement: Supplementary file 5 [file Table3.docx]

| **Parameters** | **D0 (median)** | **M3 (median)** | **M12 (median)** | **β (SE)**  **D0-M3** | **P-value (D0-M3)** | **β (SE)**  **D0-M12** | **P-value (D0-M12)** | **β (SE)**  **M3-M12** | **P-value (M3-M12)** |
| --- | --- | --- | --- | --- | --- | --- | --- | --- | --- |
| FEC_ALPHA^a^ | 3,280 | 3,306 | 3,213 | 0,06 (0,06) | 0,337 | -0,07 (0,10) | 0,475 | -0,14 (0,09) | 0,126 |
| OR_ALPHA | 3,242 | 3,139 | 3,000 | 0,00 (0,06 | 0,983 | -0,13 (0,07) | 0,068 | -0,13 (0,07) | 0,064 |
| FEC_FB_RATIO | 0,480 | 0,565 | 0,416 | 0,09 (0,05) | 0,106 | -0,02 (0,06) | 0,762 | -0,10 (0,06) | 0,071 |
| OR_FB_RATIO | 1,548 | 2,392 | 2,821 | 1,16 (0,70) | 0,104 | 1,71 (0,73) | 0,024 | 0,55 (0,73) | 0,454 |
| FEC_BACT | 0,400 | 0,408 | 0,417 | -0,02 (0,02) | 0,361 | -0,02 (0,02) | 0,437 | 0,00 (0,02) | 0,938 |
| FEC_FAFV | 0,197 | 0,239 | 0,185 | 0,04 (0,02) | 0,025 | -0,01 (0,02) | 0,557 | -0,05 (0,02) | 0,008 |
| FEC_PROT | 0,375 | 0,352 | 0,408 | -0,02 (0,02) | 0,246 | 0,03 (0,02) | 0,16 | 0,04 (0,02) | 0,015 |
| OR_BACT | 0,228 | 0,173 | 0,158 | -0,04 (0,02) | 0,035 | -0,07 (0,02) | **0,002** | -0,02 (0,02) | 0,223 |
| OR_FAFV | 0,418 | 0,427 | 0,405 | 0,03 (0,02) | 0,156 | 0,02 (0,02) | 0,389 | -0,01 (0,02) | 0,622 |
| OR_PROT | 0,340 | 0,322 | 0,406 | 0,01 (0,02) | 0,639 | 0,05 (0,02) | 0,039 | 0,04 (0,02) | 0,098 |

*Median values represent relative abundance of total in case of the different phyla. P-values, β and SE (standard error) represent results of the linear mixed model analysis.
Bold type indicates statistical significance after correction for multiple testing.*

*a Final Hessian matrix not positive definite (different model used)

Abbreviations: D0 = baseline, M3 and M12 = follow-up after 3 and 12 months. FEC = fecal samples, OR = oral samples, ALPHA = alpha diversity, FB_RATIO = Firmicutes/Bacteroidetes ratio, BACT = phylum Bacteroidetes, FAFV = phylum Firmicutes, Actinobacteria, Fusobacteria, Verrucomicrobia, PROT = phylum Proteobacteria.*
