# Supplementary material for: The gut-brain-axis one year after treatment with cladribine tablets in patients with relapsing remitting multiple sclerosis: a pilot study
Source: Front Immunol. 2025 Feb 27;16:1514762. doi: 10.3389/fimmu.2025.1514762 (PMC11903281; doi:10.3389/fimmu.2025.1514762)
Supplement: Supplementary file 6 [file Table4.docx]

*Supplementary Table 4. Differences in baseline characteristics based on response status.*

| Characteristics | Responders (n=10) | Nonresponders (n=12) | P-value |
| --- | --- | --- | --- |
| Age, years, median (IQR) | 42 (32-53) | 38 (33-47) | 0.312 |
| Female, n (%) | 7 (70) | 7 (58.3) | 0.675 |
| Body mass index, median (IQR) | 25.5 (23.5-29.0) | 26.5 (21-32.5) | 0.684 |
| Disease duration, years, median (IQR) | 7.5 (5.5-12.0) | 8.5 (2-14) | 0.947 |
| Documented relapses in past 2 years, median (IQR) | 0 (0-1) | 1 (0-1) | 0.821 |
| IMT used in last 6 months, n (%)   Glatiramer acetate  Dimethylfumarate  Teriflunomide  Fingolimod  Ocrelizumab  None | 3 (30)  2 (20)  3 (30)  1 (10)  1 (10)  0 (0) | 1 (8.3)  5 (41.7)  1 (8.3)  3 (25)  0 (0)  2 (16.7) | 0.209 |
| Reason for switch to/start of cladribine tablets, n (%)  Disease activity  Adverse events | 7 (70)  3 (30) | 9 (75)  3 (25) | 0.583 |
| Use of systemic corticosteroids in previous month, n (%) | 0 (0) | 1 (8.3) | 1 |
| Use of antibiotics in previous month, n (%) | 0 (0) | 2 (16.7) | 0.481 |
| Smoking, n (%)  Yes  Past  Never | 2 (20)  3 (30)  5 (50) | 2 (16.7)  6 (50)  4 (33.3) | 0.937 |
| EDSS at enrolment, median (IQR) | 2.5 (2.0-3.5) | 3.0 (2.0-4.0) | 0.628 |

*Abbreviations: IQR = interquartile range, IMT = immunomodulatory/immunosuppressing therapy, IVIG = intravenous immunoglobulins, EDSS = expanded disability status scale*
