# Supplementary material for: The gut-brain-axis one year after treatment with cladribine tablets in patients with relapsing remitting multiple sclerosis: a pilot study
Source: Front Immunol. 2025 Feb 27;16:1514762. doi: 10.3389/fimmu.2025.1514762 (PMC11903281; doi:10.3389/fimmu.2025.1514762)
Supplement: Supplementary file 7 [file Table5.docx]

|  | **Responder (n=10)** | | | **Non-responder (n=12)** | | | **P-value** | | | |
| --- | --- | --- | --- | --- | --- | --- | --- | --- | --- | --- |
| **Subsets** | **D0 (median)** | **M3 (median)** | **M12 (median)** | **D0 (median)** | **M3 (median)** | **M12 (median)** | **MWU D0** | **MWU M3** | **MWU M12** | **LMM overall** |
| ILC | 0,39 | 0,21 | 0,18 | 0,23 | 0,16 | 0,13 | 0,093 | 0,456 | 0,545 | 0,094 |
| GRAN | 0,04 | 0,11 | 0,09 | 0,11 | 0,12 | 0,28 | 0,107 | 0,722 | 0,062 | 0,305 |
| MONO_TOT | 16,68 | 31,61 | 26,03 | 24,48 | 30,00 | 24,25 | 0,123 | 0,771 | 0,968 | 0,456 |
| CD14_mono | 12,55 | 26,65 | 22,47 | 21,70 | 26,62 | 22,72 | 0,069 | 0,771 | 1,000 | 0,321 |
| CD16_mono | 3,48 | 4,75 | 3,55 | 2,45 | 5,32 | 3,58 | 0,314 | 0,974 | 0,840 | 0,619 |
| DC_TOT | 5,31 | 7,93 | 7,03 | 5,43 | 6,93 | 4,72 | 0,923 | 0,582 | 0,545 | 0,636 |
| cDC_TOT | 2,03 | 4,05 | 2,94 | 2,51 | 4,47 | 2,23 | 0,456 | 0,974 | 0,492 | 0,849 |
| cDC_1 | 0,03 | 0,13 | 0,36 | 0,07 | 0,09 | 0,35 | 0,283 | 0,381 | 0,840 | 0,444 |
| cDC_2 | 1,84 | 3,90 | 2,61 | 2,43 | 4,37 | 1,98 | 0,381 | 0,974 | 0,492 | 0,839 |
| mDCs | 2,15 | 2,72 | 2,01 | 1,85 | 2,46 | 1,20 | 0,722 | 0,080 | 0,206 | 0,669 |
| pDCs | 1,35 | 1,42 | 1,07 | 1,29 | 0,81 | 0,69 | 0,923 | 0,036 | 0,657 | 0,059 |
| B_TOT | 10,30 | 5,87 | 10,27 | 8,97 | 3,32 | 9,16 | 0,418 | 0,059 | 0,442 | 0,809 |
| B_CD27NEG_TOT | 7,79 | 5,47 | 9,33 | 6,12 | 2,93 | 8,47 | 0,456 | 0,069 | 0,442 | 0,826 |
| B_CD27NEG_1 | 6,70 | 4,14 | 7,94 | 5,31 | 2,33 | 5,19 | 0,418 | 0,059 | 0,310 | 0,792 |
| B_CD27NEG_2 | 1,11 | 0,89 | 1,29 | 1,18 | 0,35 | 1,18 | 0,722 | 0,140 | 0,600 | 0,499 |
| B_CD27POS | 2,26 | 0,36 | 0,46 | 1,81 | 0,21 | 0,59 | 0,418 | 0,821 | 0,840 | 0,759 |
| NK_TOT | 26,00 | 16,96 | 23,29 | 20,50 | 14,08 | 20,35 | 0,314 | 0,314 | 0,395 | 0,778 |
| NK56_DIM_TOT | 24,44 | 14,20 | 20,75 | 18,60 | 13,40 | 17,15 | 0,346 | 0,628 | 0,442 | 0,697 |
| NK56_DIM_57POS_TOT | 15,87 | 9,13 | 12,18 | 12,41 | 8,04 | 12,11 | 0,628 | 0,872 | 0,840 | 0,592 |
| NK56_DIM_57POS_1 | 11,34 | 5,24 | 8,17 | 9,68 | 6,20 | 6,64 | 0,628 | 0,539 | 0,600 | 0,726 |
| NK56_DIM_57POS_2 | 4,47 | 1,83 | 4,18 | 3,09 | 2,44 | 4,01 | 0,140 | 0,771 | 0,840 | 0,556 |
| NK56_DIM_57NEG | 8,27 | 6,08 | 7,38 | 6,60 | 4,62 | 4,83 | 0,107 | 0,203 | 0,238 | 0,889 |
| NK56_BR | 2,55 | 1,98 | 1,51 | 1,68 | ,89 | 1,42 | 0,228 | 0,254 | 0,442 | 0,293 |
| T_TOT | 33,56 | 32,05 | 32,75 | 34,93 | 38,94 | 35,76 | 0,821 | 0,418 | 0,657 | 0,603 |
| NK_T_TOT | 0,23 | 0,25 | 0,71 | 0,22 | 0,49 | 0,91 | 0,674 | 0,254 | 0,717 | 0,190 |
| GD_T_TOT | 1,89 | 2,93 | 3,51 | 2,26 | 3,32 | 3,96 | 0,923 | 0,872 | 0,904 | 0,435 |
| GD_T_57POS | 1,49 | 2,45 | 2,73 | 1,88 | 2,84 | 3,02 | 1,000 | 0,872 | 0,778 | 0,515 |
| GD_T_57NEG | 0,46 | 0,52 | 0,75 | 0,40 | 0,63 | 0,76 | 0,346 | 1,000 | 0,545 | 0,345 |
| CD4_TOT | 12,68 | 12,97 | 15,12 | 16,81 | 10,84 | 16,89 | 0,346 | 0,821 | 0,840 | 0,487 |
| CD4_TCM_TOT | 2,19 | 1,99 | 2,55 | 2,79 | 1,71 | 2,27 | 0,254 | 0,628 | 0,840 | 0,136 |
| CD4_TCM_CCR6POS | 1,71 | 1,67 | 1,88 | 2,31 | 1,42 | 1,37 | 0,539 | 0,582 | 0,968 | 0,160 |
| CD4_TCM_CXCR5POS | 0,32 | 0,34 | 0,52 | 0,81 | 0,55 | 0,59 | 0,021 | 0,456 | 0,600 | 0,163 |
| CD4_NAIVE | 3,49 | 2,00 | 4,67 | 7,07 | 1,32 | 2,71 | 0,254 | 0,722 | 0,238 | 0,037 |
| CD4_TEMRA | 0,45 | 0,21 | 0,16 | 0,36 | 0,95 | 1,00 | 0,674 | 0,283 | 0,492 | 0,385 |
| CD4_TEM_TOT | 3,59 | 2,60 | 4,51 | 4,79 | 3,77 | 8,07 | 0,722 | 0,283 | 0,351 | 0,903 |
| CD4_TEM_CD27POS | 1,99 | 2,23 | 3,13 | 3,41 | 2,55 | 4,39 | 0,539 | 0,539 | 0,492 | 0,968 |
| CD4_TEM_CD27NEG | 0,90 | 0,59 | 0,93 | 0,60 | 0,96 | 1,48 | 0,974 | 0,539 | 0,206 | 0,327 |
| CD8_TOT | 13,34 | 9,27 | 11,26 | 12,34 | 14,75 | 12,33 | 0,821 | 0,203 | 0,442 | 0,117 |
| CD8_TEM | 2,51 | 2,17 | 2,89 | 2,19 | 2,41 | 2,47 | 1,000 | 0,497 | 0,778 | 0,565 |
| CD8_NAIVE_TOT | 0,86 | 0,38 | 1,35 | 1,34 | 0,93 | 1,25 | 0,069 | 0,346 | 0,657 | 0,140 |
| CD8_TEMRA_TOT | 8,41 | 8,26 | 7,39 | 8,15 | 8,66 | 9,29 | 0,418 | 0,497 | 0,442 | 0,058 |
| CD8_TEMRA_1 | 4,12 | 4,33 | 3,34 | 2,99 | 3,41 | 2,61 | 0,381 | 0,722 | 0,600 | 0,062 |
| CD8_TEMRA_2 | 1,91 | 1,83 | 2,16 | 1,61 | 1,76 | 2,52 | 0,582 | 0,628 | 0,492 | 0,200 |
| CD8_TEMRA_3 | 2,47 | 1,85 | 2,49 | 0,66 | 1,34 | 1,13 | 0,381 | 0,821 | 0,778 | 0,220 |
| DN_T_TOT | 0,22 | 0,22 | 0,32 | 0,18 | 0,18 | 0,14 | 0,628 | 0,872 | 0,206 | 0,460 |

*Median values represent % of total immunological cells. P-value of LMM represents the results of the linear mixed model analysis with response status as interaction term, the overall p-value of the model is displayed. Lowest P-value must be <0.001 to be statistical significant after correction for multiple testing.*

*Abbreviations: D0 = baseline, M3 and M12 = follow-up after 3 and 12 months. LMM = linear mixed model. ILC = innate lymphoid cells, GRAN = granulocytes, MONO = monocytes, TOT = total, cDC = conventional dendritic cells, mDC = myeloid dendritic cells, pDC = plasmacytoid dendritic cells, NEG = negative, POS = positive, NK = natural killer, BR = bright, NK_T = natural killer T cell, GD_T = γδ T cell, TCM = central memory T cell, TEMRA = effector memory cell re-expressing CD45RA, TEM = effector memory , DN_T = double negative T cell.*
